# Predictive Score Card in Lumbar Disc Herniation: Is It Reflective of Patient Surgical Success after Discectomy?

**DOI:** 10.1371/journal.pone.0154114

**Published:** 2016-04-21

**Authors:** Parisa Azimi, Edward C. Benzel, Ali Montazeri

**Affiliations:** 1 Functional Neurosurgery Research Center, Shahid Beheshti University of Medical Sciences, Tehran, Iran; 2 Cleveland Clinic Foundation, Department of Neurosurgery, Cleveland, Ohio, United States of America; 3 Mental Health Research Group, Health Metrics Research Centre, Iranian Institute for Health Sciences Research, ACECR, Tehran, Iran; Universita degli Studi di Palermo, ITALY

## Abstract

Does the Finneson–Cooper score reflect the true value of predicting surgical success before discectomy? The aim of this study was to identify reliable predictors for surgical success two year after surgery for patients with LDH. Prospective analysis of 154 patients with LDH who underwent single-level lumbar discectomy was performed. Pre- and post-surgical success was assessed by the Oswestry Disability Index (ODI) over a 2-year period. The Finneson-Cooper score also was used for evaluation of the clinical results. Using the ODI, surgical success was defined as a 30% (or more) improvement on the ODI score from the baseline. The ODI was considered the gold standard in this study. Finally, the sensitivity, specificity, and positive and negative predictive power of the Finneson–Cooper score in predicting surgical success were calculated. The mean age of the patients was 49.6 (SD = 9.3) years and 47.4% were male. Significant improvement from the pre- to post-operative ODI scores was observed (P < 0.001). Post-surgical success was 76.0% (n = 117). The patients’ rating on surgical success assessments by the ODI discriminated well between sub-groups of patients who differed with respect to the Finneson–Cooper score. Regarding patients’ surgical success, the sensitivity, specificity, and accuracy of the Finneson-Cooper ratings correlated with success rate. The findings indicated that the Finneson–Cooper score was reflective of surgical success before discectomy.

## Introduction

lumbar disc herniation (LDH) is a very prevalent low back pain. and it is mainly a disease of elderly [1]. Most disc herniations occur in the bottom two discs of the lumbar spine, at and just below the waist. A herniated disk can irritate nearby nerves and result in pain, numbness or weakness in leg and it can lead to defecation quality of life [1–2].

Although disectomy is an effective treatment for patients with LDH, surgical outcomes vary across patient groups. Hence, surgeons should estimate possible outcomes before surgery [3–4]. What is the best way to forecast the outcome of the LDH surgery? This is a vitally important question for patients, and physicians alike for decision making after surgery. At present, many surgeons use the Finneson-Cooper score [5], for predicting surgical success. In doing so surgeons usually complete the Finneson-Copper score for patients who are potential candidates for excision of a herniated lumbar disc. If patients obtain a certain score, then the surgeon might perform surgery hoping to achieve a good success rate with an acceptable outcome. The outcome measure for such operations are usually the improvement in functional status of patients for which there are several instruments including the Oswestry Disability Index (ODI). However, many other surgeons believe that using the Finneson-Cooper score alone is not enough for predicting surgery success and we should consider some additional clinical parameters if we wish to have a better and reliable prediction. Thus, to examine to what extent the Finneson score is reliable in predicting surgery success we decided to assess this by a selected outcome measure. We were interested to see if the Finneson score is a good predictor of outcome as measured by the Oswestry Disability Index two year after surgery. In fact we used the Finneson score as a predictor and the ODI as a gold standard outcome measure. Both instruments are introduced in details in the following sections.

## Materials and Methods

### Patients and data collection

This was a prospective study. Between 2010 and 2013, consecutive patients with clinical and radiological signs of herniation of the lumbar disc underwent surgery at clinic of a teaching hospital in Tehran, Iran. The diagnosis of LDH was made on the basis of clinical and radiographic evidence. All participants underwent a complete clinical examination for LDH including an assessment of clinical symptoms and clinical examination, and imaging studies including plain radiography, computed tomography and magnetic resonance imaging of the lumbar spine. In all cases the diagnosis was confirmed by more than one spine surgeon and surgery was performed by experienced surgeons. Only those were included in the study if underwent primary lumbar discectomy with clinical and radiological evidence of compression of the lumbar nerve roots with a single-level disc herniation. Patients were asked to fill out preoperative and follow-up questionnaires and to undergo follow-up examinations at two year after surgery. Patients who had lateral or central stenosis of spinal canal, previous spine surgery, revision discectomy were excluded. Based on at least 20% failure rate for surgery we estimated that a sample of 152 patients would be enough to have a study of 80% power at 5% significant level.

Demographics including age, gender and body-mass index (BMI), VAS associated with leg pain (mm) and VAS associated with back pain (mm) were determined. The duration of symptoms (in months), type of herniation and smoking history were assessed. Surgical success was evaluated using an the ODI [6]. For all participants, the ODI was recorded at two points in time: pre-operative and two year after surgery.

### Surgery procedure

Standard open lumbar discectomy has been used to manage LDH in patients who have persistent symptoms of the condition that did not improve with a conservative treatment [7]. We used a microsurgical techniqu. Unilateral approach and bilateral approach were considerd for lateral disc and central disc and were defined fenestration and laminotomy, respectively.

### Measures

a. The Iranian version of Oswestry Disability Index (ODI) (Version 2) was used to measure functionality. The ODI contains 10 items and its score range from 0 to 50, with higher scores indicating a worse condition. The Iranian version of the ODI questionnaire is similar to the English version of the ODI and its psychometric properties are well documented [6]. The ODI score was measured at admission and at two year after surgery. Surgical success was defined as a 30% (or more) improvement on the ODI score from the baseline [8]. However, in this study, we categorized patients into two groups: good outcomes were considered a 30% (or more) improvement on the ODI scores from the baseline, and poor outcomes were considered a less than 30% improvement on the ODI score from the baseline.

b) The Finneson-Cooper score is a lumbar disc surgery predictive score that was developed by Finneson-Cooper to assess potential candidates for excision of a herniated lumbar disc [5]. The Finneson-Cooper score range from 0 to 100. The score categorizes candidates into a 4-grade classification: good >75; fair 65–75; marginal 55–64, and poor < 55. Patients in the first two categories usually recive surgery. For the purpose of this study the Finneson-Cooper score was measured once in preoperative visit.

### Statistical analysis

To achieve the study objective sensitivity analysis was performed. As such first, patients were classified using their Finneson–Cooper score and the ODI classification. Then, the ODI was considered as the gold standard for surgical success. Finally the results obtained from the estimated and actual categorizations were compared. In fact, with respect to the actual classification for each case based on the ODI score, the estimated classifications were tested and designated as true positive, true negative, false positive, or false negative in order to calculate sensitivity, specificity, positive predictive value and accuracy for the estimated classifications [9]. In addition, analysis was performed to test how well the ODI discriminates between subgroups of patients who differed in the Finneson–Cooper score. All statistical analyses were performed using the PASW Statistics 18 Version 18 (SPSS, Inc., 2009, Chicago, IL, USA). A p-value of ≤ 0.05 was considered to be statistically significant. The reference points for this study were the date of the initial surgery. The primary end point for the statistical analysis was at least 2 year of follow-up.

### Ethics

Each participant gave informed verbal consent. Since some patients were less educated, for consistency we only asked for verbal consent. The main investigator explained the study for each participant and asked for permission. It was indicated that participation and no participation does not influence the treatment and their information will remain confidential. The Ethics Committee of Shahid Beheshti University of Medical Sciences, Tehran, Iran, approved the study and agreed with the consent procedure.

## Results

In all 172 patients were approached. Of these, 18 patients were excluded (9 patients lost to follow-up, 3 patients had recurrent disk herniations, and 6 cases had spinal cord compression and spinal anomalies). The remaining 154 patients (73 men and 81 women) were included in the study. The characteristics of patients and their scores on the Finneson–Cooper score, and the ODI are shown in Table 1.

**Table 1:** Demographic data and preoperative status of patients with lumbar disc herniation (n = 154).

Characteristics	Mean (SD)
Age (Year)	49.6 (9.3)
*Range*	29–80
Gender (Male; n, %)	73(47.4)
Smoking (n, %)	56(36.5)
Body weight (kg)	82.1(9.5)
Body-mass index (BMI)	25.4 (5.1)
**Symptoms**	
Duration of symptoms (months)	15.9 (7.4)
*Range*	1–26
VAS of leg pain (mm)	56.7 (18.4)
*Range*	15–100
VAS of back pain (mm)	52.9(23.8)
*Range*	19–100
**ODI**	
Baseline	38.3 (9.2)
At last follow-up	16.8 (11.9)
Surgical success (n, %)	117 (76.0)
Not surgical success (n, %)	37 (24.0)
**Finneson–Cooper score (n, %)**	
Good	93(60.4)
Fair	51(33.1)
Marginal	8 (5.2)
Poor	2 (1.3)
**Level of hearniation (n, %)**	
L1-L2	3 (1.9)
L2-L3	7 (4.6)
L3-L4	21(13.6)
L4-L5	74 (48.1)
L5-S1	49 (31.8)
**Type of herniation (n, %)**	
Sequestration	45 (29.2)
Transligamentous extrusion	58 (37.7)
Subligamentous extrusion	37(24.0)
Protrusion	14(9.1)

Values are mean (SD), number or percentage

Discectomy via laminotomy (n = 92) and fenestration (n = 62) were performed. No case was observed with missed level surgery. Cauda-equina syndrome occurred in 1 case (0.64%). In 1 case (0.64%) dural laceration occurred during surgery, which were repaired and no one showed CSF leakage or meningitis. No mortality was observed due to surgery.

To determine patient's surgical success, the sensitivity, specificity, and accuracy of the Finneson-Cooper were estimated separately for its four categories that are ‘good’, ‘fair’, ‘marginal’ and ‘poor’. Since the number of patients in the two last categories was small, they were treated as one category. Table 2 shows the estimations for category ‘good’ while Tables 3 and 4 presenting results for category ‘fair’ and ‘marginal plus poor’, respectively. In fact Table 2 shows that out of 93 operations for those who were categorized as ‘Good’ on the baseline Finneson-Copper classification, 80 operations were perceived successful by patients as indicated by their ODI score, demonstrating a 96.4% sensitivity for the Finneson score. For these cases only there were 4 operations that seems resulted in unsatisfactory outcomes, although the Finnesson-Cooper classification was ‘Good’. Thus from such findings as indicated in Table 2 the accuracy of operations for this group (Good classification on the Finneson-Cooper score) was 92.5%. Similarly the accuracy of patient selection for discectomy for who classified as ‘fair’ (Table 3) and ‘marginal plus poor’ (Table 4) was 84.3% and 20%, respectively.

**Table 2:** Two-by-two matrices of the relationship between the estimated satisfied and the actual satisfied (sensitivity analysis).

	**Actual satisfied** *		
**Estimated satisfied** **	*Positive*	*Negative*	**Total**
*Positive*	80 (true positive)	4 (false positive)	84
*Negative*	3 (false negative)	6 (true negative)	9
**Total**	83	10	93

* Actual satisfied: Classified based on gold-standard (the ODI score).

** Estimated satisfied: Classified based on the Finneson-Cooper ‘‘Good” grade

- Sensitivity = True positives / (True positives + False negatives) = 80/ (80+3) = 96.4%

- Specificity = True negatives / (True negatives + False positives) = 6/ (6+4) = 60.0%

- Accuracy = (True positives + True negatives) / (True positives+ False positive + False negatives +True negative) = (80+6)/ (80+4+3+6) = 92.5%

- Positive predictive value (PPV) = (True positive) / (True positive + False positive) = 80/84 = 95.2%

- Negative predictive value (NPV) = (True negative) / (True negative + False negative) = 6/9 = 66.6%

**Table 3:** Two-by-two matrices of the relationship between the estimated dissatisfied and the actual dissatisfied (sensitivity analysis).

	Actual satisfied*		
**Estimated satisfied** **	*Positive*	*Negative*	**Total**
*Positive*	35(true positive)	5 (false positive)	40
*Negative*	3 (false negative)	8 (true negative)	11
**Total**	38	13	51

* Actual satisfied: Classified based on gold-standard (the ODI score).

** Estimated satisfied: Classified based on the Finneson-Cooper ‘‘Fair” grade

- Sensitivity = True positives / (True positives + False negatives) = 35/ (35+3) = 92.1%

- Specificity = True negatives / (True negatives + False positives) = 8/ (8+5) = 61.5%

- Accuracy = (True positives + True negatives) / (True positives+ False positive + False negatives +True negative) = (35+ 8)/ (35+8+5+3) = 84.3%

- Positive predictive value (PPV) = (True positive) / (True positive + False positive) = 35/40 = 87.5%

- Negative predictive value (NPV) = (True negative) / (True negative + False negative) = 8/11 = 72.7%

**Table 4:** Two-by-two matrices of the relationship between the estimated dissatisfied and the actual dissatisfied (sensitivity analysis).

	Actual satisfied*		
**Estimated satisfied** **	*Positive*	*Negative*	**Total**
*Positive*	1(true positive)	5 (false positive)	6
*Negative*	3 (false negative)	1 (true negative)	4
**Total**	4	6	10

* Actual satisfied: Classified based on gold-standard (the ODI score).

** Estimated satisfied: Classified based on the Finneson-Cooper ‘‘Marginal+ Poor” grade

- Sensitivity = True positives / (True positives + False negatives) = 1/ (1+1) = 50.0%

- Specificity = True negatives / (True negatives + False positives) = 1/ (1+5) = 16.7%

- Accuracy = (True positives + True negatives) / (True positives+ False positive + False negatives +True negative) = (1+ 1)/ (1+1+5+3) = 20.0%

- Positive predictive value (PPV) = (True positive) / (True positive + False positive) = 1/6 = 16.6%

- Negative predictive value (NPV) = (True negative) / (True negative + False negative) = 1/4 = 25.0%

The patients’ rating on surgical success assessments by the ODI discriminated well between sub-groups of patients who differed in the Finneson–Cooper score. The results are shown in Table 5. Based on the ODI post-surgical score the success rate was 76.0% (n = 117). The mean improvement for the ODI score was 21.4±12.8 and statistically was significant (p <0.001) at two year after surgery. No significant differences were observed for post-surgical success between LDH levels.

**Table 5:** The surgical success based on ODI by Finneson–Cooper score.

	Finneson-Cooper score				
	Good	Fair	Marginal	Poor	P-Value
Patients (n, %)	93 (60.4)	51 (33.1)	8 (5.2)	2 (1.3)	< 0.001
Surgical success as indicated by the ODI (n, %)	80 (86.1)	35(68.6)	2 (25.0)	0 (0.0)	< 0.001
**P-Value**	< 0.001	< 0.001	0.87	0.92	

## Discussion

The findings from this study suggest that the Finneson-Cooper score is predictive of surgical success for LDH, based on the ODI assessments before and after discectomy. In addition our data confirmed that discectomy is a safe treatment for patients who scored ‘Good’, and ‘Fair’ on the Finneson-Cooper scale. The LDH surgery was successful in the majority of cases and it is reported that nearly 90% of patients were satisfied with the operation according to the variety of measures and the various follow-up assessments [10]. In this study, based on the ODI, post-surgical success was 76.0% at 2-years following surgery. These observations indicate that we need to develop and use trustworthy metrics to assist patient selection for surgery process [5, 10–11].

Definitive predictive metrics for surgical success for the treatment of LDH have not been forthcoming. A systematic review by White et al. [12], showed that individual radiographic and clinical features were not able to predict the likelihood of surgical intervention success rates. However, they reported that higher baseline disability, as assessed by the ODI, did correlate with surgical outcomes [12]. Another systematic review reported that patients with high levels of depression, anxiety and fear-avoidance behaviors were more likely to have poor outcomes following LDH surgery [13]. Still other studies reported that Pfirrmann’s grade [14], more laterally located discs, extrusion and protrusion herniation types, and larger fragments [15] could predict the risk of conservative treatment failure in patients with LDH. Yuan et al. showed that the spinal canal and dural sac dimensions were important predictive factors for treatment selection of lumbar disc herniation [16]. In addition, psychosocial issues, personal injury litigation, and compensation claim were well known to affect outcome in low back pain [17].

The results of the current study showed that ‘Good’ and ‘Fair’ of Finneson-Cooper score can be used to predict surgical success in patients with LDH. However, more research is needed to determine the role of other factors on LDH surgical outcome and the value for screening these factors.

We used the Finneson-Cooper score as a clinical measure for known-group comparison. The findings demonstrated that patients who differed in Finneson-Cooper classification, scored differently on the ODI score at preoperative assessment. Interestingly we found that the ODI score at preoperative was higher in ‘Good’ Finneson Cooper score group compared to those who identified as ‘fair’. The findings suggest that the Finneson-Cooper score could be regarded as a valid measure to predict postoperative outcomes in patients undergoing LDH surgery. However, during the study period, we managed 10 patients who scored ‘Marginal’ (n = 8) and ‘Poor’ (n = 2) on the Finneson-Cooper scale. Clinical outcomes for these patients showed that only two patients in ‘Marginal’ group had surgical success. Out of 10 patients, 8 had diabetes and similar to other studies were found to have a poor outcome in terms of improvement in the ODI [18–19]. However, in our study, the number of patients rating ‘Marginal’ and ‘Poor’ Finneson-Cooper score was very low. Thus, although the Finneson-Cooper score is a valid tool to predict surgical success, caution is needed in using it as a sole selection factor.

### Limitations

Although prospective, this study has limitations. First, the study is limited by the small number of patients with marginal and poor Finneson-Cooper grading. Secondly, better methods for exploring outcome prediction and identifying reliable predictors of surgical outcome after LDH surgery in long-time follow-up are needed. Thirdly, this study only includes a single institution’s experience using the ODI measures. Fourthly, due to the lack of a true gold standard for assessing the patient surgical success, certain cases may have been incorrectly classified. Hence, a standardized method for evaluation of patient surgical success is needed. Finally, different spine surgeons, possibly with varying surgical expertise and skills, performed the discectomies in this study, which might bias the clinical results. Further studies are needed to improve prediction accuracy and identify reliable predictors of surgical outcome in patients with the variety of LDH.

## Conclusion

The Finneson-Cooper score may prove to be a valid tool to predict surgical success in patients with lumbar disc herniation. However, using other relevant factors should not be neglected.

## Supporting Information

S1 File A minimal set of data for the study. (SAV)
